# Bis[2,6-bis­(3,5-dimethyl-1*H*-pyrazol-1-yl)pyridine]di-μ_3_-iodido-diiodidotetra­copper(I)

**DOI:** 10.1107/S1600536811026468

**Published:** 2011-07-09

**Authors:** Chun-Xiao Jia

**Affiliations:** a Department of Chemistry, DeZhou University, De Zhou, Shan Dong 253023, People’s Republic of China

## Abstract

In the title centrosymmetric tetra­nuclear complex, [Cu_4_I_4_(C_15_H_17_N_5_)_2_], the two distinct Cu^I^ atoms adopt similar tetra­hedral arrangements, each being ligated by two I atoms, and two N atoms from one 2,6-bis­(3,5-dimethyl-1*H*-pyrazol-1-yl)pyridine ligand. In the crystal, there are no hydrogen bonds  present, and only very weak π–π inter­actions  are observed [centroid–centroid distance = 3.985 (4) Å], which connect neighbouring tetra­nuclear units into a chain motif along the *b* axis.

## Related literature

For related structures and background references, see: Carina *et al.* (1998 ▶); Constable *et al.* (1994 ▶); Piguet *et al.* (1989 ▶); Solanki *et al.* (1999 ▶); Laza­rou *et al.* (2009 ▶, 2010 ▶). For tetra­hedral geometry, see: Halcrow *et al.* (1997 ▶).
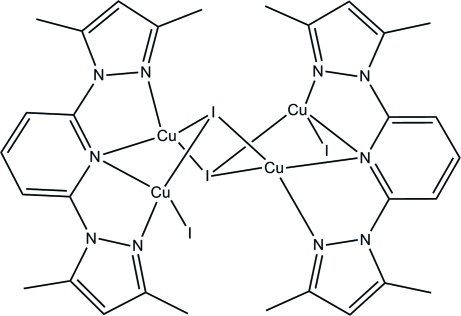


## Experimental

### 

#### Crystal data

[Cu_4_I_4_(C_15_H_17_N_5_)_2_]
*M* *_r_* = 1296.47
Monoclinic, 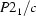

*a* = 10.196 (2) Å
*b* = 11.425 (2) Å
*c* = 16.572 (3) Å
β = 98.67 (3)°
*V* = 1908.3 (7) Å^3^
*Z* = 2
Mo *K*α radiation
μ = 5.47 mm^−1^
*T* = 296 K
0.23 × 0.20 × 0.19 mm

#### Data collection

Rigaku Mercury diffractometer
Absorption correction: multi-scan (REQAB; Jacobson, 1998 ▶) *T*_min_ = 0.366, *T*_max_ = 0.423
18181 measured reflections
3484 independent reflections
3090 reflections with *I* > 2σ(*I*)
*R*_int_ = 0.044

#### Refinement

*R*[*F* ^2^ > 2σ(*F* ^2^)] = 0.044
*wR*(*F* ^2^) = 0.144
*S* = 1.04
3484 reflections
221 parameters
H-atom parameters constrained
Δρ_max_ = 0.79 e Å^−3^
Δρ_min_ = −1.14 e Å^−3^

### 

Data collection: *CrystalClear* (Rigaku, 2007 ▶); cell refinement: *CrystalClear*; data reduction: *CrystalClear*; program(s) used to solve structure: *SHELXS97* (Sheldrick, 2008 ▶); program(s) used to refine structure: *SHELXL97* (Sheldrick, 2008 ▶); molecular graphics: *DIAMOND* (Brandenburg, 1999 ▶); software used to prepare material for publication: *SHELXTL* (Sheldrick, 2008 ▶).

## Supplementary Material

Crystal structure: contains datablock(s) I, global. DOI: 10.1107/S1600536811026468/su2284sup1.cif

Structure factors: contains datablock(s) I. DOI: 10.1107/S1600536811026468/su2284Isup2.hkl

Additional supplementary materials:  crystallographic information; 3D view; checkCIF report

## Figures and Tables

**Fig. 1.**
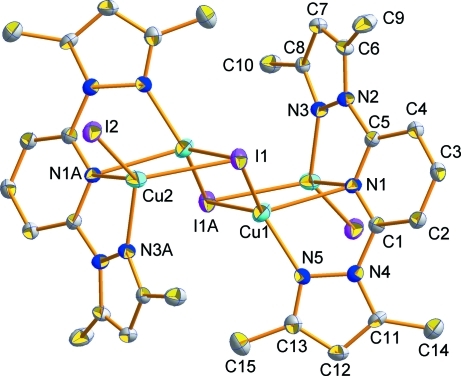
Molecular structure of the title compound, showing the numbering scheme and displacement ellipsoids drawn at the 30% probability level [Symmetry code: (a) -x+2, -y, -z].

**Fig. 2.**
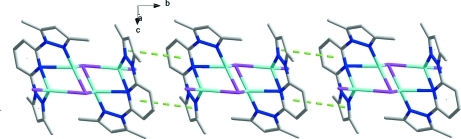
A view along the b axis of the crystal packing of the title compound. The π–π interactions are shown as green dashed lines.

## supplementary crystallographic information

### Comment

The study of dimeric double helical complexes generated from copper(I) salts and 2,6-bis(imidazol-2-yl)pyridine ligands demonstrates that, the variable copper(I) coordination geometries in the helicates can exhibit different connectivity patterns, such as {4+2}, {2+2+2}, and {3+3} motifs (Carina *et al.*, 1998; Constable *et al.*, 1994; Piguet *et al.*, 1989; Solanki *et al.*, 1999). Recently, some auxiliary ligands have also been introduced into these coordination systems (Lazarou *et al.*, 2009, 2010). Here, we used iodine ions to bridge the copper(I) ions, which resulted in the tetranuclear coordination motif of the title compound (Fig. 1).

There are two crystallographic distinct copper(I) centers in this complex, which display similar coordination environments. Each metal center is linked to two nitrogen atoms from one 2,6-bis(3,5-dimethyl-1*H*-pyrazol-1-yl)pyridine ligand, and to two iodine ions to give the tetrahedral coordination geometry. Both the geometries at atoms Cu1 and Cu2 are slightly flattened, the dihedral angles between the planes of the donors [Cu,N,N] and [Cu,I,I] are 75.2 (2)° and 81.46 (19)°, for atoms Cu1 and Cu2, respectively; they should be 90° for an 'ideal' tetrahedron (Halcrow *et al.*, 1997). Paired copper(I) atoms are ligated to one 2,6-bis(3,5-dimethyl-1*H*-pyrazol-1-yl)pyridine ligand acting as a tridentate ligand, forming two five membered chelate rings. In favour of the bridging role of the I1 donor is the fact that the binuclear coordination units, which are interlinked via an invesion center into a tetranuclear entity, feature two kinds of coordination squares with the compositions of Cu_2_IN and Cu_2_I_2_.

In the crystal, there exists a very weak π–π interaction, between the pyridyl ring (N1/C1—C5) and a pyrazol ring (N2,N3/C6—C8) from an adjacent molecule, with a centroid to centroid distance of 3.985 (4) Å and a dihedral angle of 24.72° (Fig. 2).

### Experimental

A mixture of CuI (15 mg, 0.1 mmol) and 2,6-bis(3,5-dimethyl-1*H*-pyrazol-1-yl)pyridine (13 mg, 0.05 mmol) was dissolved in MeCN (2 ml) in air. A light green solution was obtained, which was transferred into a Pyrex glass tube. It was then sealed and heated to 393 K for 48 h, and cooled to room temperature at a rate of 5 °C/h. Blue blocks of the title complex were formed. They were collected by filtration, washed with MeCN/Et_2_O (*v*/*v* = 1: 4) and dried in air.

### Refinement

The C-bound H-atoms were included in calculated positions and treated as riding atoms: C-H = 0.96 Å for CH_3_(methyl), with U_iso_(H) = 1.5U_eq_(parent C-atom).

### Crystal data

**Table:**

[Cu_4_I_4_(C_15_H_17_N_5_)_2_]	*F*(000) = 1224
*M**_r_* = 1296.47	*D*_x_ = 2.256 Mg m^−^^3^
Monoclinic, *P*2_1_/*c*	Mo *K*α radiation, λ = 0.71073 Å
Hall symbol: -P 2ybc	Cell parameters from 6904 reflections
*a* = 10.196 (2) Å	θ = 3.1–25.4°
*b* = 11.425 (2) Å	µ = 5.47 mm^−^^1^
*c* = 16.572 (3) Å	*T* = 296 K
β = 98.67 (3)°	Block, yellow
*V* = 1908.3 (7) Å^3^	0.23 × 0.20 × 0.19 mm
*Z* = 2	

### Data collection

**Table:**

Rigaku Mercury diffractometer	3484 independent reflections
Radiation source: fine-focus sealed tube	3090 reflections with *I* > 2σ(*I*)
graphite	*R*_int_ = 0.044
ω scans	θ_max_ = 25.4°, θ_min_ = 3.1°
Absorption correction: multi-scan (REQAB; Jacobson, 1998)	*h* = −12→12
*T*_min_ = 0.366, *T*_max_ = 0.423	*k* = −13→13
18181 measured reflections	*l* = −18→19

### Refinement

**Table:**

Refinement on *F*^2^	Primary atom site location: structure-invariant direct methods
Least-squares matrix: full	Secondary atom site location: difference Fourier map
*R*[*F*^2^ > 2σ(*F*^2^)] = 0.044	Hydrogen site location: inferred from neighbouring sites
*wR*(*F*^2^) = 0.144	H-atom parameters constrained
*S* = 1.04	*w* = 1/[σ^2^(*F*_o_^2^) + (0.1*P*)^2^] where *P* = (*F*_o_^2^ + 2*F*_c_^2^)/3
3484 reflections	(Δ/σ)_max_ = 0.001
221 parameters	Δρ_max_ = 0.79 e Å^−^^3^
0 restraints	Δρ_min_ = −1.14 e Å^−^^3^

### Special details

**Table:**

Geometry. Bond distances, angles etc. have been calculated using the rounded fractional coordinates. All su's are estimated from the variances of the (full) variance-covariance matrix. The cell esds are taken into account in the estimation of distances, angles and torsion angles
Refinement. Refinement of *F*^2^ against ALL reflections. The weighted *R*-factor *wR* and goodness of fit *S* are based on *F*^2^, conventional *R*-factors *R* are based on *F*, with *F* set to zero for negative *F*^2^. The threshold expression of *F*^2^ > σ(*F*^2^) is used only for calculating *R*-factors(gt) *etc*. and is not relevant to the choice of reflections for refinement. *R*-factors based on *F*^2^ are statistically about twice as large as those based on *F*, and *R*- factors based on ALL data will be even larger.

### Fractional atomic coordinates and isotropic or equivalent isotropic displacement parameters (Å^2^)

**Table:**

	*x*	*y*	*z*	*U*_iso_*/*U*_eq_	
I1	0.89222 (5)	0.01954 (4)	0.10655 (3)	0.0451 (2)	
I2	0.69949 (5)	−0.31427 (5)	0.14285 (3)	0.0560 (2)	
Cu1	1.09482 (9)	0.06814 (8)	0.03673 (5)	0.0496 (3)	
Cu2	0.88858 (8)	−0.21963 (8)	0.09155 (6)	0.0505 (3)	
N1	1.0760 (5)	0.2853 (4)	0.0443 (3)	0.0330 (16)	
N2	0.8750 (5)	0.3195 (4)	−0.0368 (3)	0.0317 (16)	
N3	0.9228 (5)	0.2702 (4)	−0.1017 (3)	0.0327 (16)	
N4	1.2814 (5)	0.2399 (4)	0.1183 (3)	0.0370 (17)	
N5	1.2690 (5)	0.1243 (4)	0.0920 (3)	0.0390 (17)	
C1	1.1655 (6)	0.3094 (5)	0.1094 (4)	0.0338 (17)	
C2	1.1460 (6)	0.3916 (5)	0.1671 (4)	0.0373 (19)	
C3	1.0338 (7)	0.4591 (5)	0.1528 (4)	0.0394 (19)	
C4	0.9396 (6)	0.4392 (5)	0.0853 (4)	0.0353 (17)	
C5	0.9650 (6)	0.3487 (5)	0.0339 (3)	0.0329 (17)	
C6	0.7411 (6)	0.3407 (6)	−0.0557 (4)	0.0385 (19)	
C7	0.7065 (6)	0.3060 (6)	−0.1340 (4)	0.0392 (19)	
C8	0.8209 (6)	0.2622 (5)	−0.1604 (4)	0.0364 (17)	
C9	0.6551 (7)	0.3812 (8)	0.0042 (5)	0.061 (3)	
C10	0.8402 (7)	0.2080 (7)	−0.2401 (4)	0.050 (2)	
C11	1.4084 (6)	0.2670 (6)	0.1455 (4)	0.0409 (19)	
C12	1.4805 (7)	0.1673 (6)	0.1367 (5)	0.050 (2)	
C13	1.3894 (7)	0.0821 (6)	0.1042 (4)	0.046 (2)	
C14	1.4556 (8)	0.3854 (7)	0.1752 (5)	0.059 (3)	
C15	1.4186 (10)	−0.0425 (7)	0.0865 (6)	0.074 (3)	
H2	1.20710	0.40110	0.21430	0.0450*	
H3	1.02100	0.51880	0.18890	0.0470*	
H4	0.86290	0.48410	0.07480	0.0420*	
H7	0.62240	0.31040	−0.16460	0.0470*	
H9A	0.64980	0.46510	0.00300	0.0910*	
H9B	0.69250	0.35580	0.05800	0.0910*	
H9C	0.56790	0.34870	−0.01000	0.0910*	
H10A	0.93010	0.21970	−0.24900	0.0750*	
H10B	0.78090	0.24390	−0.28360	0.0750*	
H10C	0.82190	0.12560	−0.23880	0.0750*	
H12	1.57190	0.15840	0.14980	0.0610*	
H14A	1.40540	0.44440	0.14280	0.0890*	
H14B	1.54790	0.39370	0.17060	0.0890*	
H14C	1.44390	0.39430	0.23130	0.0890*	
H15A	1.37580	−0.09290	0.12080	0.1100*	
H15B	1.51270	−0.05530	0.09690	0.1100*	
H15C	1.38620	−0.05940	0.03030	0.1100*	

### Atomic displacement parameters (Å^2^)

**Table:**

	*U*^11^	*U*^22^	*U*^33^	*U*^12^	*U*^13^	*U*^23^
I1	0.0518 (3)	0.0381 (3)	0.0481 (3)	−0.0022 (2)	0.0159 (2)	−0.0044 (2)
I2	0.0392 (3)	0.0522 (3)	0.0810 (4)	−0.0087 (2)	0.0229 (3)	−0.0150 (2)
Cu1	0.0423 (5)	0.0509 (5)	0.0544 (5)	−0.0101 (4)	0.0032 (4)	−0.0077 (4)
Cu2	0.0319 (5)	0.0540 (6)	0.0667 (6)	0.0032 (4)	0.0112 (4)	0.0004 (4)
N1	0.027 (3)	0.038 (3)	0.034 (2)	0.000 (2)	0.005 (2)	−0.003 (2)
N2	0.027 (3)	0.037 (3)	0.032 (2)	0.000 (2)	0.007 (2)	0.001 (2)
N3	0.030 (3)	0.036 (3)	0.033 (2)	0.001 (2)	0.008 (2)	0.003 (2)
N4	0.035 (3)	0.036 (3)	0.039 (3)	−0.002 (2)	0.002 (2)	−0.003 (2)
N5	0.034 (3)	0.031 (3)	0.051 (3)	0.003 (2)	0.003 (2)	0.002 (2)
C1	0.033 (3)	0.034 (3)	0.035 (3)	0.000 (3)	0.007 (3)	0.003 (3)
C2	0.040 (4)	0.036 (3)	0.035 (3)	−0.005 (3)	0.003 (3)	0.001 (3)
C3	0.044 (4)	0.031 (3)	0.045 (3)	0.000 (3)	0.013 (3)	−0.003 (3)
C4	0.037 (3)	0.032 (3)	0.037 (3)	0.000 (3)	0.006 (3)	0.000 (3)
C5	0.031 (3)	0.031 (3)	0.036 (3)	−0.004 (3)	0.003 (3)	−0.003 (2)
C6	0.029 (3)	0.039 (3)	0.049 (4)	0.006 (3)	0.011 (3)	−0.004 (3)
C7	0.031 (3)	0.039 (4)	0.045 (3)	0.002 (3)	−0.003 (3)	0.001 (3)
C8	0.033 (3)	0.031 (3)	0.044 (3)	0.002 (3)	0.002 (3)	−0.009 (3)
C9	0.033 (4)	0.085 (6)	0.066 (5)	−0.003 (4)	0.012 (4)	−0.018 (4)
C10	0.047 (4)	0.064 (5)	0.038 (3)	0.009 (4)	0.001 (3)	−0.010 (3)
C11	0.029 (3)	0.048 (4)	0.043 (3)	−0.009 (3)	−0.003 (3)	−0.005 (3)
C12	0.031 (4)	0.052 (4)	0.066 (4)	0.004 (3)	0.000 (3)	0.006 (4)
C13	0.045 (4)	0.039 (4)	0.052 (4)	−0.003 (3)	0.005 (3)	0.006 (3)
C14	0.049 (5)	0.054 (5)	0.072 (5)	−0.011 (4)	0.002 (4)	−0.008 (4)
C15	0.073 (6)	0.046 (5)	0.101 (7)	0.011 (4)	0.010 (5)	−0.001 (5)

### Geometric parameters (Å, °)

**Table:**

I1—Cu1	2.5758 (12)	C7—C8	1.399 (9)
I1—Cu2	2.7435 (12)	C8—C10	1.498 (9)
I1—Cu1^i^	2.5988 (11)	C11—C12	1.375 (10)
I2—Cu2	2.4704 (11)	C11—C14	1.494 (11)
Cu1—N1	2.493 (5)	C12—C13	1.396 (10)
Cu1—N5	1.978 (5)	C13—C15	1.493 (11)
Cu2—N1^i^	2.451 (5)	C2—H2	0.9300
Cu2—N3^i^	1.991 (5)	C3—H3	0.9300
N1—C1	1.333 (8)	C4—H4	0.9300
N1—C5	1.333 (8)	C7—H7	0.9300
N2—N3	1.367 (7)	C9—H9A	0.9600
N2—C5	1.415 (7)	C9—H9B	0.9600
N2—C6	1.375 (8)	C9—H9C	0.9600
N3—C8	1.315 (8)	C10—H10A	0.9600
N4—N5	1.391 (7)	C10—H10B	0.9600
N4—C1	1.413 (8)	C10—H10C	0.9600
N4—C11	1.342 (8)	C12—H12	0.9300
N5—C13	1.306 (9)	C14—H14A	0.9600
C1—C2	1.376 (9)	C14—H14B	0.9600
C2—C3	1.370 (9)	C14—H14C	0.9600
C3—C4	1.379 (9)	C15—H15A	0.9600
C4—C5	1.389 (8)	C15—H15B	0.9600
C6—C7	1.352 (9)	C15—H15C	0.9600
C6—C9	1.494 (10)		
			
Cu1—I1—Cu2	100.07 (4)	C6—C7—C8	107.2 (6)
Cu1—I1—Cu1^i^	61.18 (4)	N3—C8—C7	110.3 (6)
Cu1^i^—I1—Cu2	62.30 (4)	N3—C8—C10	119.1 (6)
I1—Cu1—N1	96.83 (13)	C7—C8—C10	130.6 (6)
I1—Cu1—N5	125.92 (15)	N4—C11—C12	106.1 (6)
I1—Cu1—I1^i^	118.82 (5)	N4—C11—C14	124.5 (6)
N1—Cu1—N5	74.01 (18)	C12—C11—C14	129.4 (6)
I1^i^—Cu1—N1	116.39 (12)	C11—C12—C13	106.5 (6)
I1^i^—Cu1—N5	112.48 (15)	N5—C13—C12	111.0 (6)
I1—Cu2—I2	113.91 (5)	N5—C13—C15	122.2 (7)
I1—Cu2—N1^i^	112.72 (12)	C12—C13—C15	126.9 (7)
I1—Cu2—N3^i^	106.36 (14)	C1—C2—H2	121.00
I2—Cu2—N1^i^	114.38 (13)	C3—C2—H2	121.00
I2—Cu2—N3^i^	129.64 (15)	C2—C3—H3	120.00
N1^i^—Cu2—N3^i^	73.41 (19)	C4—C3—H3	120.00
Cu1—N1—C1	101.4 (4)	C3—C4—H4	122.00
Cu1—N1—C5	127.2 (4)	C5—C4—H4	122.00
Cu1—N1—Cu2^i^	68.04 (13)	C6—C7—H7	126.00
C1—N1—C5	117.2 (5)	C8—C7—H7	126.00
Cu2^i^—N1—C1	129.0 (4)	C6—C9—H9A	109.00
Cu2^i^—N1—C5	106.7 (3)	C6—C9—H9B	109.00
N3—N2—C5	119.0 (5)	C6—C9—H9C	109.00
N3—N2—C6	110.7 (5)	H9A—C9—H9B	110.00
C5—N2—C6	130.1 (5)	H9A—C9—H9C	110.00
N2—N3—C8	106.0 (5)	H9B—C9—H9C	110.00
Cu2^i^—N3—N2	120.5 (4)	C8—C10—H10A	109.00
Cu2^i^—N3—C8	133.5 (4)	C8—C10—H10B	109.00
N5—N4—C1	117.8 (5)	C8—C10—H10C	109.00
N5—N4—C11	111.2 (5)	H10A—C10—H10B	109.00
C1—N4—C11	130.9 (5)	H10A—C10—H10C	109.00
Cu1—N5—N4	119.1 (4)	H10B—C10—H10C	109.00
Cu1—N5—C13	135.2 (4)	C11—C12—H12	127.00
N4—N5—C13	105.3 (5)	C13—C12—H12	127.00
N1—C1—N4	115.4 (5)	C11—C14—H14A	109.00
N1—C1—C2	123.4 (6)	C11—C14—H14B	110.00
N4—C1—C2	121.2 (6)	C11—C14—H14C	110.00
C1—C2—C3	117.9 (6)	H14A—C14—H14B	109.00
C2—C3—C4	120.6 (6)	H14A—C14—H14C	109.00
C3—C4—C5	116.7 (6)	H14B—C14—H14C	109.00
N1—C5—N2	114.3 (5)	C13—C15—H15A	110.00
N1—C5—C4	124.0 (5)	C13—C15—H15B	109.00
N2—C5—C4	121.7 (5)	C13—C15—H15C	109.00
N2—C6—C7	105.9 (5)	H15A—C15—H15B	110.00
N2—C6—C9	124.4 (6)	H15A—C15—H15C	109.00
C7—C6—C9	129.4 (6)	H15B—C15—H15C	109.00
			
Cu2—I1—Cu1—N1	175.69 (12)	C1—N1—C5—N2	179.3 (5)
Cu1^i^—I1—Cu1—N1	125.23 (12)	C1—N1—C5—C4	1.8 (9)
Cu2—I1—Cu1—N5	−109.13 (18)	Cu2^i^—N1—C5—N2	26.1 (5)
Cu1^i^—I1—Cu1—N5	−159.60 (18)	Cu2^i^—N1—C5—C4	−151.4 (5)
Cu2—I1—Cu1—I1^i^	50.47 (5)	C5—N2—N3—C8	−174.6 (5)
Cu1^i^—I1—Cu1—I1^i^	0.02 (10)	C5—N2—N3—Cu2^i^	8.5 (6)
Cu1^i^—I1^i^—Cu1—I1	0.02 (11)	C6—N2—N3—C8	0.8 (6)
Cu2^i^—I1^i^—Cu1—I1	120.94 (6)	C6—N2—N3—Cu2^i^	−176.1 (4)
Cu1^i^—I1^i^—Cu1—N1	−115.12 (14)	N3—N2—C5—N1	−25.8 (7)
Cu2^i^—I1^i^—Cu1—N1	5.83 (14)	N3—N2—C5—C4	151.8 (5)
Cu1^i^—I1^i^—Cu1—N5	162.21 (16)	C6—N2—C5—N1	159.9 (6)
Cu2^i^—I1^i^—Cu1—N5	−76.85 (15)	C6—N2—C5—C4	−22.5 (9)
Cu1—I1—Cu2—I2	−176.47 (5)	N3—N2—C6—C7	−1.2 (7)
Cu1^i^—I1—Cu2—I2	−126.72 (6)	N3—N2—C6—C9	172.7 (6)
Cu1—I1—Cu2—N1^i^	−44.00 (14)	C5—N2—C6—C7	173.5 (6)
Cu1^i^—I1—Cu2—N1^i^	5.75 (14)	C5—N2—C6—C9	−12.7 (11)
Cu1—I1—Cu2—N3^i^	34.49 (16)	N2—N3—C8—C7	−0.1 (7)
Cu1^i^—I1—Cu2—N3^i^	84.24 (16)	N2—N3—C8—C10	−178.4 (5)
N1—Cu1—N5—C13	−159.0 (6)	Cu2^i^—N3—C8—C7	176.3 (4)
I1^i^—Cu1—N5—N4	124.0 (4)	Cu2^i^—N3—C8—C10	−2.0 (9)
I1^i^—Cu1—N5—C13	−46.6 (6)	C1—N4—N5—Cu1	4.6 (7)
I1—Cu1—N1—C1	99.2 (4)	C1—N4—N5—C13	177.7 (5)
I1—Cu1—N1—C5	−38.4 (5)	C11—N4—N5—Cu1	−172.7 (4)
I1—Cu1—N1—Cu2^i^	−133.19 (9)	C11—N4—N5—C13	0.5 (7)
N5—Cu1—N1—C1	−26.3 (4)	N5—N4—C1—N1	−33.3 (7)
N5—Cu1—N1—C5	−163.9 (5)	N5—N4—C1—C2	143.6 (6)
N5—Cu1—N1—Cu2^i^	101.33 (18)	C11—N4—C1—N1	143.3 (6)
I1^i^—Cu1—N1—C1	−133.9 (3)	C11—N4—C1—C2	−39.8 (10)
I1^i^—Cu1—N1—C5	88.5 (5)	N5—N4—C11—C12	0.1 (7)
I1^i^—Cu1—N1—Cu2^i^	−6.23 (15)	N5—N4—C11—C14	177.7 (6)
I1—Cu1—N5—N4	−75.3 (4)	C1—N4—C11—C12	−176.7 (6)
I1—Cu1—N5—C13	114.2 (6)	C1—N4—C11—C14	1.0 (11)
N1—Cu1—N5—N4	11.6 (4)	Cu1—N5—C13—C12	170.7 (5)
I1—Cu2—N1^i^—Cu1^i^	−5.73 (14)	Cu1—N5—C13—C15	−10.8 (10)
I1—Cu2—N1^i^—C1^i^	−92.8 (5)	N4—N5—C13—C12	−0.8 (7)
I1—Cu2—N1^i^—C5^i^	118.3 (3)	N4—N5—C13—C15	177.7 (6)
I2—Cu2—N1^i^—Cu1^i^	126.52 (8)	N1—C1—C2—C3	−6.0 (9)
I2—Cu2—N1^i^—C1^i^	39.5 (5)	N4—C1—C2—C3	177.4 (6)
I2—Cu2—N1^i^—C5^i^	−109.5 (4)	C1—C2—C3—C4	4.3 (9)
I1—Cu2—N3^i^—N2^i^	−114.1 (4)	C2—C3—C4—C5	−0.1 (9)
I1—Cu2—N3^i^—C8^i^	61.9 (6)	C3—C4—C5—N1	−3.2 (9)
I2—Cu2—N3^i^—N2^i^	103.6 (4)	C3—C4—C5—N2	179.4 (5)
I2—Cu2—N3^i^—C8^i^	−80.5 (6)	N2—C6—C7—C8	1.1 (7)
Cu1—N1—C1—N4	36.9 (6)	C9—C6—C7—C8	−172.4 (7)
Cu1—N1—C1—C2	−139.9 (5)	C6—C7—C8—N3	−0.6 (8)
C5—N1—C1—N4	179.8 (5)	C6—C7—C8—C10	177.4 (7)
C5—N1—C1—C2	3.0 (9)	N4—C11—C12—C13	−0.6 (8)
Cu2^i^—N1—C1—N4	−34.0 (7)	C14—C11—C12—C13	−178.1 (7)
Cu2^i^—N1—C1—C2	149.3 (5)	C11—C12—C13—N5	0.9 (8)
Cu1—N1—C5—N2	−48.7 (6)	C11—C12—C13—C15	−177.5 (7)
Cu1—N1—C5—C4	133.8 (5)		

Symmetry codes: (i) −*x*+2, −*y*, −*z*.
